# Impact of blood pressure on the outcomes of inpatients with Subarachnoid hemorrhage: A retrospective cross-sectional study

**DOI:** 10.1097/MD.0000000000024761

**Published:** 2021-02-19

**Authors:** Naohito Saito, Tetsuo Nishikawa, Tetsuo Ota

**Affiliations:** a Department of Physical Medicine and Rehabilitation, Sunagawa City Medical Center, Sunagawa, Hokkaido; b Endocrinology & Diabetes Center, Yokohama Rosai Hospital, Yokohama, Kanagawa; c Department of Physical Medicine and Rehabilitation, Asahikawa Medical University Hospital, Asahikawa, Hokkaido, Japan.

**Keywords:** activities of daily living, hypertension, inpatients, stroke, subarachnoid hemorrhage

## Abstract

It is unclear whether antihypertensive treatment should be indicated after subarachnoid hemorrhage (SAH). Hence, we investigated the impact of blood pressure on inpatient outcomes after SAH rehabilitation.

This retrospective cross-sectional study analyzed data of SAH inpatients, as obtained from the Japan Association of Rehabilitation Database for inpatients undergoing SAH rehabilitation. Inpatients admitted to a conventional ward with a diagnosis of cerebrovascular disease were voluntarily registered in this database between January 2006 and December 2013 from hospitals in Japan. Patients were categorized into hypertensive and non-hypertensive populations and assessed using the Barthel Index (BI) and the total BI score at hospital discharge. We compared the independent population (patients with the highest score for each activity) with its non-independent counterpart. Data on the patients’ age, BI score on admission, total BI score, BI score increase, daily BI score increase, hospitalization duration, BI activities, patients’ sex, and Brunnstrom recovery stage were compared.

Eighty-eight patients with SAH were analyzed; 43 were hypertensive and 45 non-hypertensive. Hypertension was associated with increased non-independence levels (hypertensive versus non-hypertensive patients, transfers [bed to chair and back]: 15 versus 24, *P* = .03, odds ratio (OR) = 2.532 (95% confidence interval [CI], 1.065–6.024); toilet use: 15 versus 24, *P* = .03, OR = 2.532 (95% CI, 1.065–6.024); bathing: 23 versus 34, *P* = .0061, OR = 3.623 (95% CI, 1.414–9.259); stair climbing: 22 versus 31, *P* = .03, OR = 2.703 (95% CI, 1.114–6.579); and bladder control: 14 versus 24, *P* = .02, OR = 2.801 (95% CI, 1.170–6.711)). The total BI score of the hypertensive inpatients at discharge was lower than that of their non-hypertensive counterparts (0–75 versus 80–100, 30 versus 19, *P* = .03). Moreover, the BI score increase per day was significantly lower in the hypertensive group than in the non-hypertensive group (.67 versus 1.8, *P* = .02). The hypertensive group also had a significantly longer duration of hospitalization than the hypertensive group (52 versus 30 days, *P* = .02).

Hypertension was associated with longer hospitalization and poorer outcomes post-discharge, suggesting the importance of strict blood pressure control in patients who have experienced SAH.

## 1 Introduction

Subarachnoid hemorrhage (SAH) is a significant cause of morbidity and mortality, worldwide. In a systematic review of studies published in October 2005, De Rooij et al found that the overall incidence of SAH was 9.1 per 100,000 person-years.^[1]^ In Japan and Finland, the incidence was shown to be higher than that in other countries, at 22.7 and 19.7 per 100,000, respectively. The outcomes after aneurysmal SAH depend on the severity of the initial hemorrhage, occurrence of rebleeding, perioperative medical management, and the timing and technical success of aneurysm exclusion from the cerebral circulation. Important modifiable risk factors for SAH are cigarette smoking, hypertension, and excessive alcohol intake, whereas non-modifiable risk factors include sex, age, aneurysm size, and family history.^[2]^ Juvela investigated 142 patients with 181 unruptured aneurysms diagnosed between 1956 and 1978 and followed-up the patients until death, SAH development, or 1997 to 2000, and found that increased systolic blood pressure (BP) values and long-term hypertension before aneurysm rupture predict fatal SAH independently of aneurysm size, patient sex, or age at the time of rupture.^[3]^ A review by Feigin et al revealed that hypertension increased the risk of SAH by nearly 2.5-fold.^[4]^

The Barthel Index (BI) is a measure of patient independence that provides a score indicating the ability of a person with a neuromuscular or musculoskeletal disorder to care for himself/herself,^[5]^ and it is also a highly valuable scoring system for the prognostic prediction of patients with acute cerebral infarction.^[6]^ The BI evaluates 10 basic activities of self-care (feeding, grooming, dressing, toilet use, bathing, control of bowels, and bladder control) and mobility (transfers [bed to chair and back], walking on a level surface, and stair climbing) with a total score ranging from 0 (total dependence) to 100 (totally independent functioning). As the BI does not include an assessment of death, it is not translatable to all 6 levels of the modified Rankin scale (mRS), which is another dependency index. The BI possesses certain advantages in the assessment of the activities of daily living (ADLs), which include completeness, sensitivity to change, reliability, suitability for statistical manipulation, and greater familiarity owing to widespread use.^[7–10]^

Considering that hypertension is readily treatable and that data on the impact of BP on SAH recovery are limited, we performed this study to analyze the effect of BP on the outcomes of patients post-SAH rehabilitation using the BI in Japan.

## 2 Materials and methods

### 2.1 Ethical considerations

We analyzed a deidentified dataset in this study; therefore, the requirement for written informed consent from the patients was waived. Our study was approved (#2019-35) by the ethical advisory committee of Sunagawa City Medical Center and was conducted ethically in accordance with the World Medical Association Declaration of Helsinki.

### 2.2 Data source

**Figure 1:**
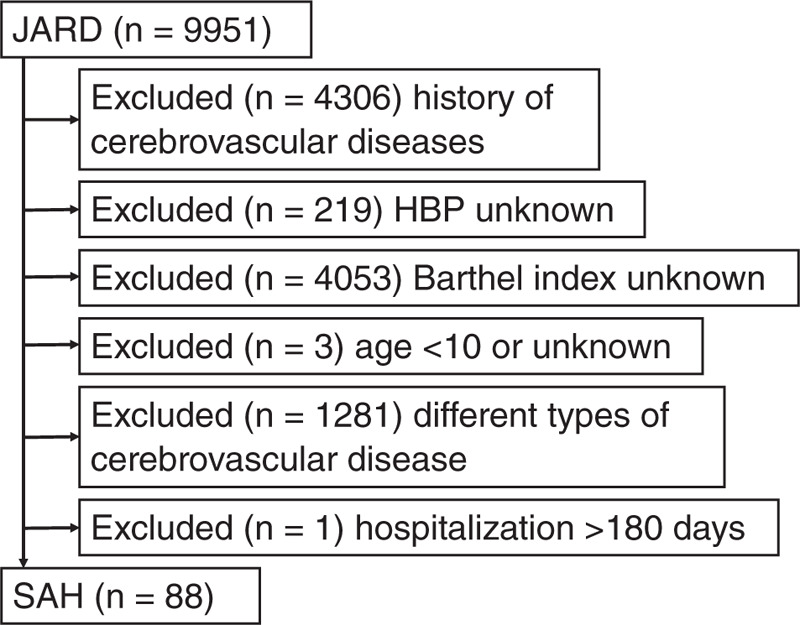
Flow chart showing the selection of inpatients with subarachnoid hemorrhage. HBP = high blood pressure (hypertension), JARD = Japan Association of Rehabilitation Database, SAH = subarachnoid hemorrhage.

In this retrospective cross-sectional study, we analyzed SAH inpatient data, as obtained from the Japan Association of Rehabilitation Database (JARD; the data can be obtained from http://square.umin.ac.jp/JARD/index.html). Inpatients admitted to a conventional ward with a diagnosis of cerebrovascular disease (lacuna infarction, atherothrombotic infarction, cardioembolic infarction, infarction of other types, hypertensive hemorrhage, hemorrhage of other types, and SAH) were observed until hospital discharge, and voluntarily registered in this database between January 2006 and December 2013 from hospitals in Japan. We included patients with no medical history of cerebrovascular diseases, those with complete records on hypertension and BI, those aged 10 or older, those with SAH, and those hospitalized for ≤180 days. Finally, 88 patients were included in the statistical analysis (Fig. 1). We divided them to compare the data between hypertensive patients and their non-hypertensive counterparts.

### 2.3 Hypertension

Hypertension was diagnosed and categorized in each hospital. It was defined as 2 consecutive systolic BP measurements ≥140 mm Hg and/or diastolic BP levels ≥90 mm Hg, according to the general definition used in Japan. Patients taking antihypertensive medications were categorized as having hypertension regardless of their BP measurements.

### 2.4 BI

The BI was assessed at hospital admission and discharge; the value at admission is a potential bias of ADL outcomes, while that at discharge is an outcome. We divided each BI activity into the independent population (the highest score for each activity) versus the non-independent population. We also divided the total BI scores into 0 to 75 (BI 80-) versus 80 to 100, 0–55 (BI 60-) versus 60 to 100, 0–35 (BI 40-) versus 40 to 100, or 0 to 15 (BI 20-) versus 20 to 100 to compare each category and its respective control. The total BI scores were also divided by the number of hospitalization days to calculate the daily change in BI.

### 2.5 Brunnstrom recovery stage (BRS)

Paresis is associated with potential bias in ADL outcomes. BRS is reflective of the impact of hypertension on paresis of the upper extremities, the hands and fingers, and the lower extremities, given that it plays a role in patients’ health status deterioration after stroke.^[11]^ In our study, BRS was categorized into 1 versus 2–6, 1–2 versus 3–6, 1–3 versus 4–6, 1–4 versus 5–6, and 1–5 versus 6.

### 2.6 Statistical analysis

Data on the patients’ age, BI score on admission, total BI score, BI score increase, daily BI score increase, and hospitalization duration were compared using Student *t*-test (Wilcoxon test) for continuous variables. Total BI score, BI activities, patients’ sex, and BRS were compared using the Pearson chi-square test (Fisher exact test) for non-continuous variables by drawing a 2 × 2 contingency table. Statistical analysis was performed with JMP10 software (SAS Institute, Inc., Cary, NC) and *P*-values < .05 were considered significant.

## 3 Results

Figure 1 shows the data selection procedure employed in our study. The JARD included 9,951 cerebrovascular disease inpatients admitted to conventional wards.

The hypertensive patients showed significantly higher levels of non-independence at hospital discharge for transfers than the non-hypertensive patients (bed to chair and back, 15 versus 24, *P* = .03), toilet use (15 versus 24, *P* = .03), bathing (23 versus 34, *P* = .0061), stair climbing (22 versus 31, *P* = .03), and bladder control (14 versus 24, *P* = .02) (Table 1). The total BI score in the hypertensive inpatients, as obtained using ordinal analysis, was significantly lower than that in their non-hypertensive counterparts (BI score: 0–75 vs. 80–100, *P* = .03). We analyzed the BI scores in both the hypertensive and non-hypertensive patients with continuous analysis; the median values were 60 and 95, respectively (*P* = .01). The median BI score was 0 for both hypertensive and non-hypertensive inpatients at hospital admission; the difference was not statistically significant (*P* = .16) (Table 2) when analyzed under the assumption that they are continuous variables. The duration of hospitalization was significantly longer in the hypertensive inpatients than in the non-hypertensive inpatients (52 versus 30 days, *P* = .02). The increase in BI score during hospitalization (again, with the caveat that they were treated as continuous variables) was not significantly different between the hypertensive and non-hypertensive patients (45 versus 60, *P* = .11), although the daily BI score increase was significantly lower in the former than in the latter group (.67 versus 1.8, *P* = .02).

**Table 1:** Effect of hypertension on the outcomes of inpatients with subarachnoid hemorrhage after stroke rehabilitation.

	BI	Score	Non-HBP (n = 45)	HBP (n = 43)	*P*-value
BI activities	Feeding	0 and 5 vs. 10	13	17	.29
	Transfers	0, 5, and 10 vs. 15	15	24	.03∗
	Grooming	0 vs. 5	13	18	.20
	Toilet use	0 and 5 vs. 10	15	24	.03∗
	Bathing	0 vs. 5	23	34	.0061∗
	Walking	0, 5, and 10 vs. 15	39	32	.15
	Stairs	0 and 5 vs. 10	22	31	.03∗
	Dressing	0 and 5 vs. 10	40	38	.94
	Bowel care	0 and 5 vs. 10	14	21	.09
	Bladder	0 and 5 vs. 10	14	24	.02∗
BI score	80-	0–75 vs. 80–100	30	19	.03∗
	60-	0–55 vs. 60–100	31	22	.09
	40-	0–35 vs. 40–100	33	27	.29
	20-	0–15 vs. 20–100	37	30	.17

**Table 2:** Number, age, Barthel index scores, and hospitalization durations of inpatients with subarachnoid hemorrhage.

	Non-HBP	HBP	*P*-value
Patients, n	45	43	.79
Male, n	18	16	
Female, n	27	27	
Age, years	63	61	.83
BI score at admission	0	0	.16
BI score increase	60	45	.11
Daily BI score increase	1.8	.67	.02∗
Duration of hospitalization, days	30	52	.02∗

Finally, the analysis of the available paresis data on 72 inpatients who experienced SAH, including 33 hypertensive and 39 non-hypertensive patients, revealed that hypertension did not affect the BRS (Table 3). Notably, no significant differences in sex or age were observed between the hypertensive and non-hypertensive inpatients (Table 2).

**Table 3:** Effect of hypertension on Brunnstrom Recovery Stage scores.

BRS		Score	Non-HBP (n = 37)	HBP (n = 27)	*P*-value
UE	1	1 vs 2-6	0	1	.45
	2	1-2 vs 3-6	3	3	1.0
	3	1-3 vs 4-6	4	3	1.0
	4	1-4 vs 5-6	7	4	.74
	5	1-5 vs 6	8	5	.59
HF	1	1 vs 2-6	0	1	.45
	2	1-2 vs 3-6	3	3	1.0
	3	1-3 vs 4-6	4	3	1.0
	4	1-4 vs 5-6	6	3	.50
	5	1-5 vs 6	8	4	.52
LE	1	1 vs 2-6	0	1	.45
	2	1-2 vs 3-6	2	2	1.0
	3	1-3 vs 4-6	4	2	.68
	4	1-4 vs 5-6	5	4	1.0
	5	1-5 vs 6	7	5	.80

## 4 Discussion

Our study is unique in that BI activities were used to analyze the impact of hypertension on patients’ post-SAH ADLs. We found that hypertension was associated with a deterioration in several BI activities. A lower total BI score at the time of hospital discharge was also confirmed among the hypertensive inpatients. To the best of our knowledge, no previous studies have investigated whether the duration of hospitalization is longer among hypertensive inpatients who have experienced SAH.

In the present study, individual BI activities were analyzed using the Pearson chi-square test or Fisher exact test, as they are categorical variables; thus, our statistical methodology is appropriate. This analysis allowed the consideration of BI activities as meaningful measures of ADL, thereby demonstrating that this method may serve as a useful alternative to the more commonly used total BI. Using a continuous scale for the BI score would be inappropriate, as this score is the sum of individual BI activities (ordinary variables). Therefore, in the present study, we divided the BI scores into categories for statistical analysis. The obtained results were statistically significant; thus, BI activities have some advantages over the BI in the assessment of the ADLs of people with cerebrovascular diseases, elderly people, and the general population.

In the present study, no significant differences were observed between the hypertensive and non-hypertensive inpatients in terms of BI activities, including feeding, grooming, walking, dressing, and bowel care. Three of these items (with the exception of walking) are self-care items and may require a lower circulatory system load than the mobility-related items. Conversely, the deteriorations in the quality of transfers, toilet use, bathing, stair climbing, and bladder care in the hypertensive inpatients, all of which require mobility skills that may exert a heavier load on the circulatory system, were statistically significant. These BI activity results show that hypertension leads to ADL deteriorations at hospital discharge after SAH rehabilitation.

The proportion of hypertensive inpatients whose total BI score was ≥80 was significantly lower than that of non-hypertensive inpatients. Granger et al found that 75% of patients with stroke and BI scores of at least 85 could independently perform chair and toilet transfers, while more than one-third could walk 50 yards independently.^[12]^ A score of 85 usually corresponds to independence with minimal assistance. Inpatients whose BI scores are ≥80 are, therefore, likely to be predominantly independent, except when climbing/descending stairs and bathing, and can be discharged from the hospital. We found that a greater proportion of non-hypertensive inpatients could be discharged than their hypertensive counterparts. The deterioration of the BI bathing score (*P* = .0061) among the hypertensive inpatients may have strongly affected the total BI score at hospital discharge. The impact of hypertension on ADL after stroke rehabilitation has been previously studied, predominantly using the mRS as the outcome measure.^[13–17]^ However, we posit that ADL assessment, using the BI combined with mRS mortality estimation, may provide a more appropriate measure of outcomes than other methods (potentially including neuroimaging).

Inamasu et al performed a retrospective study to evaluate whether hypertension, as observed in the emergency department, is predictive of poor outcomes in patients with SAH using the mRS at 30 days after admission^[18]^ and concluded that an initial systolic BP ≥220 mm Hg may be a crude indicator of poor outcomes in these patients. Ohkuma et al investigated 273 Asian patients with SAH who were admitted to Japanese institutions within 24 hours of their initial bleeding and found that a systolic arterial pressure >160 mm Hg was a possible risk factor for rebleeding.^[19]^ They also studied 198 patients with aneurysmal SAH between 1989 and 1998 and found that a history of hypertension increased the risk of SAH.^[20]^ Rosengart et al analyzed data of 3,567 patients with aneurysmal SAH who participated in 4 randomized, double-blind, placebo-controlled clinical trials of tirilazad^[21]^ and found that a high systolic BP at admission and a history of hypertension were associated with unfavorable outcomes at 3 months after aneurysmal SAH surgical treatment. The American Heart Association/American Stroke Association guidelines recommend oral nimodipine administration for the management of cerebral vasospasms and delayed cerebral ischemia after aneurysmal SAH for all patients.^[22]^ These guidelines emphasize that the agent improves neurological outcomes, but not the rates of cerebral vasospasm, as well as that, while the magnitude of BP control required for the reduction of the risk of rebleeding is not established, a decrease in the systolic BP to <160 mm Hg may be reasonable in preventing rebleeding after SAH. Further, the induction of hypertension for patients with delayed cerebral ischemia is recommended. Our results are compatible with those of most previous studies that reported on SAH and encourage the implementation of strict BP control following current guidelines.

Our study has some limitations. First, a systematic protocol for the recording of BP was not available, and we relied on the measurements acquired during routine clinical practice at each hospital. It was not possible to observe the dose-dependent effect of BP on rehabilitation outcomes, as the BP readings were divided into the hypertensive and non-hypertensive types; individual readings were not available. Further, data on the effect of BP variations were not available. This is an important area for future research. Second, as the hypertensive inpatients were supposedly treated with antihypertensives following existing guidelines, and as the impact of hypertension could have been observed more reliably in patients who were not taking these agents, the presence of bias cannot be ruled out. Third, our data were obtained based on the results of our previous analysis on Asians—predominantly Japanese people; and none of the analyzed patients were Africans or Caucasians. Finally, we did not assess mortality, which may have biased our study, although this has already been investigated by another group.^[18]^ Despite these limitations, our results encourage the implementation of strict BP control among patients admitted to the hospital for rehabilitation after SAH.

Our study also has several strengths. First, our analysis excluded inpatients with a history of stroke; only those with primary SAH were analyzed. Second, paresis and ADL at hospital admission and discharge were assessed by those who were involved with rehabilitation medicine using BRS and the BI. Third, precise assessments and analyses performed using the total BI score and individual BI activities aided in the observation of the impact of BP on ADL after SAH rehabilitation. Lastly, we investigated the duration of hospitalization after SAH.

## 5 Conclusions

Hypertension was associated with longer durations of hospital stay among patients with SAH and a deterioration in the BI combined with a slower daily BI score increase during hospitalization. Although longer hospitalization durations should be prospectively evaluated with a larger number of demographic features in future studies, we believe that strict BP control can improve patients’ rehabilitation outcomes after SAH, worldwide.

## Acknowledgments

We thank the members of the Japan Association of Rehabilitation Database (JARD), who established and managed the database and gathered the data in collaboration with the investigators at the clinics and other study units. Without them, the performance of this study would not have been possible. The content is solely the responsibility of the authors and does not necessarily represent the official views of the JARD.

## Author contributions

**Data curation:** Naohito Saito.

**Formal analysis:** Naohito Saito.

**Methodology:** Naohito Saito, Tetsuo Ota.

**Project administration:** Naohito Saito.

**Resources:** Naohito Saito.

**Supervision:** Tetsuo Nishikawa, Tetsuo Ota.

**Writing – original draft:** Naohito Saito.

**Writing – review and editing:** Tetsuo Nishikawa, Tetsuo Ota.

## References

[1] de Rooij NK Linn FHH van der Plas JA. Incidence of subarachnoid haemorrhage: a systematic review with emphasis on region, age, gender and time trends. J Neurol Neurosurg Psychiatry 2007;78:1365–72. 17470467 10.1136/jnnp.2007.117655 PMC2095631

[2] Steiner T Juvela S Unterberg A. European Stroke Organization guidelines for the management of intracranial aneurysms and subarachnoid haemorrhage. Cerebrovasc Dis 2013;35:93–112. 23406828 10.1159/000346087

[3] Juvela S. Prehemorrhage risk factors for fatal intracranial aneurysm rupture. Stroke 2003;34:1852–7. 12829865 10.1161/01.STR.0000080380.56799.DD

[4] Feigin V Parag V Lawes CMM. Smoking and elevated blood pressure are the most important risk factors for subarachnoid hemorrhage in the Asia-Pacific region: an overview of 26 cohorts involving 306,620 participants. Stroke 2005;36:1360–5. 15933249 10.1161/01.STR.0000170710.95689.41

[5] Mahoney FI Barthel DW. Functional evaluation: the Barthel index. Md State Med J 1965;14:61–5. 14258950

[6] Li Q-X Zhao X-J Wang Y. Value of the Barthel scale in prognostic prediction for patients with cerebral infarction. BMC Cardiovasc Disord 2020;20:14. 31931720 10.1186/s12872-019-01306-1 PMC6956477

[7] Kwon S Hartzema AG Duncan PW. Disability measures in stroke: relationship among the Barthel Index, the Functional Independence Measure, and the Modified Rankin Scale. Stroke 2004;35:918–23. 14976324 10.1161/01.STR.0000119385.56094.32

[8] Gresham GE Phillips TF Labi ML. ADL status in stroke: relative merits of three standard indexes. Arch Phys Med Rehabil 1980;61:355–8. 7406673

[9] Wolfe CD Taub NA Woodrow EJ. Assessment of scales of disability and handicap for stroke patients. Stroke 1991;22:1242–4. 1833860 10.1161/01.str.22.10.1242

[10] Quinn TJ Langhorne P Stott DJ. Barthel index for stroke trials: development, properties, and application. Stroke 2011;42:1146–51. 21372310 10.1161/STROKEAHA.110.598540

[11] Brunnstrom S. Motor testing procedures in hemiplegia: based on sequential recovery stages. Phys Ther 1966;46:357–75. 5907254 10.1093/ptj/46.4.357

[12] Granger CV Dewis LS Peters NC. Stroke rehabilitation: analysis of repeated Barthel index measures. Arch Phys Med Rehabil 1979;60:14–7. 420565

[13] Anderson CS Heeley E Huang Y. Rapid blood-pressure lowering in patients with acute intracerebral hemorrhage. N Engl J Med 2013;368:2355–65. 23713578 10.1056/NEJMoa1214609

[14] Butcher KS Jeerakathil T Hill M. The intracerebral hemorrhage acutely decreasing arterial pressure trial. Stroke 2013;44:620–6. 23391776 10.1161/STROKEAHA.111.000188

[15] He J Zhang Y Xu T. Effects of immediate blood pressure reduction on death and major disability in patients with acute ischemic stroke: the CATIS randomized clinical trial. JAMA 2014;311:479–89. 24240777 10.1001/jama.2013.282543

[16] Lee M Ovbiagele B Hong K-S. Effect of blood pressure lowering in early ischemic stroke: meta-analysis. Stroke 2015;46:1883–9. 26022636 10.1161/STROKEAHA.115.009552

[17] Qureshi AI Palesch YY Barsan WG. Intensive blood-pressure lowering in patients with acute cerebral hemorrhage. N Engl J Med 2016;375:1033–43. 27276234 10.1056/NEJMoa1603460 PMC5345109

[18] Inamasu J Oheda M Ito K. Relationship between systolic blood pressures measured in emergency department and outcomes in patients with subarachnoid hemorrhage. Acute Med Surg 2015;2:35–9. 29123688 10.1002/ams2.63 PMC5667197

[19] Ohkuma H Tsurutani H Suzuki S. Incidence and significance of early aneurysmal rebleeding before neurosurgical or neurological management. Stroke 2001;32:1176–80. 11340229 10.1161/01.str.32.5.1176

[20] Ohkuma H Tabata H Suzuki S. Risk factors for aneurysmal subarachnoid hemorrhage in Aomori, Japan. Stroke 2003;34:96–100. 12511757 10.1161/01.str.0000048161.57536.42

[21] Rosengart AJ Schultheiss KE Tolentino J. Prognostic factors for outcome in patients with aneurysmal subarachnoid hemorrhage. Stroke 2007;38:2315–21. 17569871 10.1161/STROKEAHA.107.484360

[22] Connolly ES Jr Rabinstein AA Carhuapoma JR. Guidelines for the management of aneurysmal subarachnoid hemorrhage: a guideline for healthcare professionals from the american heart association/american stroke association. Stroke 2012;43:1711–37. 22556195 10.1161/STR.0b013e3182587839

